# Intraspecific phylogeny and genomic resources development for an important medical plant *Dioscorea nipponica*, based on low-coverage whole genome sequencing data

**DOI:** 10.3389/fpls.2023.1320473

**Published:** 2023-12-12

**Authors:** Ke Hu, Min Chen, Pan Li, Xiaoqin Sun, Ruisen Lu

**Affiliations:** ^1^ Institute of Botany, Jiangsu Province and Chinese Academy of Sciences, Nanjing, China; ^2^ Jiangsu Key Laboratory for the Research and Utilization of Plant Resources, Nanjing, China; ^3^ Laboratory of Systematic & Evolutionary Botany and Biodiversity, College of Life Sciences, Zhejiang University, Hangzhou, China; ^4^ Jiangsu Provincial Science and Technology Resources Coordination Platform (Agricultural Germplasm Resources) Germplasm Resources Nursery of Medicinal Plants, Nanjing, China

**Keywords:** *Dioscorea nipponica*, plastome-derived markers, polymorphic nSSRs, SCNGs, intraspecific phylogeny

## Abstract

*Dioscorea nipponica* Makino, a perennial twining herb with medicinal importance, has a disjunctive distribution in the Sino-Japanese Floristic Region. It has a long history in traditional Chinese medicine, with demonstrated efficacy against various health conditions. However, the limited genomic data and knowledge of genetic variation have hindered its comprehensive exploration, utilization and conservation. In this study, we undertook low-coverage whole genome sequencing of diverse *D. nipponica* accessions to develop both plastome (including whole plastome sequences, plastome-derived SSRs and plastome-divergent hotspots) and nuclear genomic resources (including polymorphic nuclear SSRs and single-copy nuclear genes), as well as elucidate the intraspecific phylogeny of this species. Our research revealed 639 plastome-derived SSRs and highlighted six key mutational hotspots (namely CDS *ycf1*, IGS *trnL*-*rpl32*, IGS *trnE*-*trnT*, IGS *rps16*-*trnQ*, Intron 1 of *clpP*, and Intron *trnG*) within these accessions. Besides, three IGS regions (i.e., *ndhD*-*cssA*, *trnL*-*rpl32*, *trnD*-*trnY*), and the intron *rps16* were identified as potential markers for distinguishing *D. nipponica* from its closely related species. In parallel, we successfully developed 988 high-quality candidate polymorphic nuclear SSRs and identified 17 single-copy nuclear genes for *D. nipponica*, all of which empower us to conduct in-depth investigations into phylogenetics and population genetics of this species. Although our phylogenetic analyses, based on plastome sequences and single-copy nuclear genes revealed cytonuclear discordance within *D. nipponica*, both findings challenged the current subspecies classification. In summary, this study developed a wealth of genomic resources for *D. nipponica* and enhanced our understanding of the intraspecific phylogeny of this species, offering valuable insights that can be instrumental in the conservation and strategic utilization of this economically significant plant.

## 1 Introduction

*Dioscorea nipponica* Makino, a perennial twining herb of the genus *Dioscorea* belonging to the family Dioscoreaceae, is disjunctively distributed across the Sino-Japanese Floristic Region (Obidiegwu et al., 2020; Yang et al., 2022). This plant is characterized by its slender and cylindrical aerial stem, alternate simple palmate leaves with an anisometric triangular shallow, medium, or deep crack along the leaf edge, and unisexual yellowish-green flowers that droop like small bells (Ding and Gilbert, 2000; Ou-Yang et al., 2018). As it matures, it produces dry capsules that are yellow, obovate-elliptic, and prismatic in shape with winged edges (Ding and Gilbert, 2000). *Dioscorea nipponica* has a long history of use in traditional Chinese medicine, where it has proven effective against various conditions, including rheumatoid arthritis, Kashin-Beck disease, sprains, bruises, chronic bronchitis, and cough (Ou-Yang et al., 2018; Yang et al., 2022). Modern pharmacological research has also revealed the multifaceted attributes of *D. nipponica*, showcasing its wide-ranging benefits, such as its anti-inflammatory, anti-tumor, analgesic, antitussive, calming, and phlegm-dispelling properties (Wu et al., 2023). Notably, recent scientific studies have isolated both fat-soluble and water-soluble steroidal saponins from rhizomes of *D. nipponica*, attributing most of its pharmacological effects to saponins and sapogenins (Ou-Yang et al., 2018). Additionally, its aboveground parts contain over ten types of phenanthrene derivatives, further contributing to its medicinal value (Lu et al., 2010; Li et al., 2017). Despite its medicinal significance, a pivotal research gap lies in the limited availability of genomic information, impeding the exploration of new bioactive compounds and a comprehensive understanding of their synthesis. Furthermore, although demand is increasing with growing awareness, the development and utilization of *D. nipponica* resources have been slow (Ou-Yang et al., 2018). The species faces challenges meeting market demand due to the gradual depletion and unrestrained exploitation of wild resources (Chen et al., 2007; Ou-Yang et al., 2018). Therefore, there is an urgent need for additional molecular markers to enhance efforts related to conservation, utilization, and breeding of this economically significant species.

To date, the taxonomic classification and intraspecific phylogenetic relationship of *D. nipponica* have remained subjects of ongoing debate and controversy. Various studies have proposed different perspectives, leading to conflicting perspectives on the subspecies delineation. For instance, Ding and Gilbert (2000) proposed a division of *D. nipponica* into two distinct subspecies, drawing upon considerations such as chromosome count, cork layer characteristics, and geographical distribution. The original subspecies, *D. nipponica* subsp. *nipponica*, is predominantly located in the northern reaches of the Qinling Mountain range, characterized by a rhizome with an easily peelable cork layer and a chromosome count of 20. In contrast, *D. nipponica* subsp. *rosthornii*, the second subspecies, features a persistent cork layer and a chromosome count of 40, primarily inhabiting the southern region of the Qinling Mountains in central China. However, Gao et al. (2008) presented an alternative perspective, suggesting a closer relationship between *D. nipponica* subsp. *rosthornii* and *D. althaeoides* than with *D. nipponica* subsp. *nipponica*. Their proposition advocated for the elevation of *D. nipponica* subsp. *rosthornii* to the status of an independent species, a viewpoint further corroborated by the chemotaxonomic analysis conducted by Li et al. (2020). Our recent phylogenetic analysis of *Stenophora* species/subspecies based on complete plastome sequences provided strong support for the monophyly of *D. nipponica* subsp. *nipponica* and *D. nipponica* subsp. *rosthornii* (Hu et al., 2023). Nevertheless, it is crucial to acknowledge that all previous studies have been limited by their restricted taxon sampling, typically involving just one specimen each for *D. nipponica* subsp. *nipponica* and *D. nipponica* subsp. *rosthornii* (e.g., Gao et al., 2008; Hu et al., 2023), or relying on a limited set of genetic markers, such as *matK*, *rbcL*, and *trnL-F* (e.g., Gao et al., 2008). Therefore, obtaining a more extensive range of diverse samples from various geographic regions is crucial, with a specific focus on covering both the northern and southern areas of the Qinling Mountains and expanding the scope beyond China’s borders. Additionally, employing stronger molecular markers, such as both plastomes and multiple nuclear loci, is vital for conducting intraspecific phylogenetic analysis of this species.

The advent of next-generation sequencing (NGS) technologies has ignited a profound revolution in the acquisition of genome-scale data across a broad spectrum of plant species (Dodsworth, 2015; Lu et al., 2022). Within the realm of NGS methodologies, low-coverage whole genome sequencing, commonly known as genome skimming, has emerged as a notably cost-effective and efficient approach (Straub et al., 2012; Twyford and Ness, 2017; Jin et al., 2020). This technique employs shallow sequencing of genomic DNA, strategically capturing a significant fraction of the genome, with a particular emphasis on high-copy elements such as ribosomal DNA, the plastome, and nuclear repeats like simple sequence repeats (SSRs) and transposable elements (Straub et al., 2012; Dodsworth, 2015; Nevill et al., 2020; Lu et al., 2022). Plastome sequences, among these molecular markers, have proven immensely valuable in plant species identification and phylogenetic studies, thanks to their distinctive characteristics: the absence of recombination, low rates of nucleotide substitutions, small effective population sizes, and typically uniparental inheritance (Birky et al., 1983; Lu et al., 2021). Moreover, recent studies have demonstrated the capacity to recover single-copy nuclear genes (SCNGs) by leveraging multiple assembled nuclear sequences derived from low-coverage whole genome sequencing data (e.g., Liu et al., 2021; Zhou et al., 2022), offering a promising resource for plant phylogenetic analyses.

Thus, to expand genomic resources available for *D. nipponica* and deepen our insights into its intraspecific phylogeny and taxonomy, we conducted low-coverage whole genome sequencing on diverse *D. nipponica* accessions originating from various regions, including both the northern and southern areas of the Qinling Mountains, as well as Japan. Our objectives encompassed the following key aspects: 1) identification of plastome-derived markers, including whole plastome sequences, plastome-derived SSRs and plastome-divergent hotspots; 2) development of genome-wide nuclear markers, encompassing polymorphic nuclear SSRs (PolynSSRs) and SCNGs; and 3) reconstruction of the phylogenetic relationships among *D. nipponica* accessions based on both plastome and SCNG data. These findings will offer valuable support for the conservation and strategic utilization of this economically significant species.

## 2 Materials and methods

### 2.1 Plant materials, DNA extraction and genome sequencing

A sample of eight *D. nipponica* accessions originating from diverse regions, including Beijing (BJ), Gansu (GS), Henan (HeN), Hunan (HuN), Jining (JN) and Zhejiang (ZJ) Provinces of China, as well as Fukushima (FD) and Nagano (NI) Prefectures of Japan was used in this study (**Table 1**). The sampling strategy was designed to encompass the primary geographical distribution of this species, including both the northern and southern areas of the Qinling Mountains, as well as Japan, and was carried out under the special permission granted by the Institute of Botany, Jiangsu Province and Chinese Academy of Sciences. For each accession, fresh, healthy leaf samples were collected from a wild mature individual, and dried with silica-gel. The voucher specimens were deposited at Herbarium of Institute of Botany, Jiangsu Province and Chinese Academy of Sciences (NAS). Genomic DNA was extracted from approximately 50 mg of silica-dried leaf samples using DNAsecure Plant Kit (Tiangen Biotech, Beijing, China), in accordance with the manufacturer’s prescribed protocol. DNA quantity and purity were checked by spectrophotometry and agarose gel electrophoresis.

**Table 1:** Summary of characteristics of eight *Dioscorea nipponica* plastomes.

Characteristics	BJ	GS	HeN	HuN	JN	ZJ	FD	NI
Locality	Beijing, China	Gansu, China	Henan, China	Hunan, China	Jining, China	Zhejiang, China	Fukushima, Japan	Nagano, Japan
Latitude (N)/Longitude (E)	40°02’/115°28’	35°33’/106°31’	35°10’/112°35’	29°24’/110°29’	41°56’/126°24	30°11’/119°33	37°38’/140°05’	35°50’/137°54
Total clean reads	30,324,374	31,352,778	23,737,170	24,959,536	32,495,028	31,790,950	33,758,516	28,825,392
Mapped reads	2,699,436	885,479	937,679	1,206,815	463,218	742,377	791,711	553,670
Plastome coverage (×)	2631	863	914	1176	451	723	771	539
Total plastome length (bp)	153,917	153,918	153,917	153,918	153,918	153,963	154,076	154,074
LSC	83,982	83,983	83,982	83,983	83,983	84,028	84,108	84,103
SSC	18,919	18,919	18,919	18,919	18,919	18,919	18,908	18,911
IR	25,508	25,508	25,508	25,508	25,508	25,508	25,530	25,530
Total GC content (%)	37.20%	37.20%	37.20%	37.20%	37.20%	37.20%	37.20%	37.20%
LSC	35.00%	35.00%	35.00%	35.00%	35.00%	35.00%	35.00%	35.00%
SSC	31.20%	31.20%	31.20%	31.20%	31.20%	31.20%	31.30%	31.30%
IR	43.00%	43.00%	43.00%	43.00%	43.00%	43.00%	43.00%	43.00%
Total number of genes	114	114	114	114	114	114	114	114
PCGs	80	80	80	80	80	80	80	80
tRNA genes	30	30	30	30	30	30	30	30
rRNA genes	4	4	4	4	4	4	4	4
Duplicated genes	19	19	19	19	19	19	19	19

For each accession, a barcoded paired-end library with an insert size of 350 bp was constructed using NEBNext Ultra DNA Library Prep Kit for Illumina. Following the ligation of indexed adapters, these indexed DNA libraries were pooled and subjected to paired-end sequencing in a single lane of HiSeq X Ten. Subsequently, Trimmomatic v.0.36 (Bolger et al., 2014) was employed to eliminate adaptor sequences, contamination, and low-quality reads from the raw data. This filtering process yielded approximately 4.5 Gb of clean data for each accession. The entire sequence library preparation, genome sequencing, and raw data filtering procedures were conducted by Novogene Bioinformatics Technology Co., Ltd., located in Beijing, China.

### 2.2 Plastome assembly and annotation

The clean reads from each accession were utilized for plastome sequence assembly using GetOrganelle v.1.7.6 (Jin et al., 2020), with the following parameters: -R 15 -k 21,45,65,85,105 -F embplant_pt. Subsequently, all assembly graphs underwent thorough visual examination using Bandage v.0.8.1 (Wick et al., 2015). To further assess the precision of plastome assemblies, two methods were employed including mapping Illumina reads back to the plastome sequence of each *D. nipponica* accession using CLC Genomics Workbench v.23.0.4 (https://digitalinsights.qiagen.com), and comparing our assemblies to NOVOPlasty (Dierckxsens et al., 2017) assemblies. The initial annotation of the newly assembled plastomes was conducted with MAFFT v.7 plugin (Katoh and Standley, 2013) within Geneious Prime® 2022.0.1 (https://www.geneious.com). This annotation involved aligning the newly assembled plastomes to the previously published plastome of *D. nipponica* (OQ525997), and transferring reference annotations to these newly assembled plastomes. Following this initial annotation, a meticulous manual verification was conducted to ensure the accuracy of exon/intron boundaries and the precise locations of start/stop codons. Finally, high-resolution circular plastome maps were generated using the web-based tool OrganellarGenomeDRAW (OGDRAW) v.1.3.1 (Greiner et al., 2019).

### 2.3 Characterization of plastome-derived SSRs

The MISA-web application (Beier et al., 2017) was utilized to identify simple sequence repeats (SSRs) within the eight newly assembled plastome sequences of *D. nipponica*. SSR identification criteria were as follows: a minimum threshold of 10 repeat units for mononucleotide SSRs, 5 for dinucleotide SSRs, 4 for trinucleotide SSRs, and 3 for tetra-, penta-, and hexanucleotide SSRs, respectively. Data visualization was performed by the OmicStudio tools at https://www.omicstudio.cn/tool (Lyu et al., 2023).

### 2.4 Identification of plastome-divergent hotspots

To identify mutational hotspots in *D. nipponica* plastomes, three steps were undertaken: firstly, aligning these eight newly assembled plastome sequences using the MAFFT v.7 plugin (Katoh and Standley, 2013) in Geneious Prime® 2022.0.1; secondly, manually extracting coding regions (CDS), introns, and intergenic spacers (IGS) that met specific criteria: (a) the total number of mutation > 0; and (b) an aligned length >200 bp; and finally, subjecting all extracted regions to DnaSP v.6.12.03 (Rozas et al., 2017) for calculating nucleotide diversity (*π*).

To further develop potential molecular markers for distinguishing *D. nipponica* from its closely related species, i.e., *D. collettii*, *D. gracillima*, *D. villosa*, and *D. zingiberensis* (Hu et al., 2023), a total of six plastome sequences, including two for *D. nipponica* (BJ and FD, representing Chinese and Japanese accession, respectively), and one each for *D. collettii* (OQ525992), *D. gracillima* (OQ525995), *D. villosa* (KY085893), and *D. zingiberensis* (OQ526000) were analyzed. Nucleotide diversity of CDS, introns and IGS regions was computed using the same method described above.

### 2.5 Development of polymorphic nuclear SSRs

Low-coverage whole genome sequencing data from each *D. nipponica* accession were firstly mapped onto the genome sequences of *D. alata* (Bredeson et al., 2022) and *D. zingiberensis* (Li et al., 2022) to exclude mitochondria and chloroplast reads, using BWA-MEM v.0.7.17 (Li, 2013). The resulting Binary Alignment/Map (BAM) data, which exclusively contained nuclear reads, were *de novo* assembled into scaffolds using the SOAPdenovo2 program (Luo et al., 2012), a de Bruijn graph-based assembly program. Subsequent to nuclear scaffolds generation, the identification of candidate polymorphic nuclear SSRs (PolynSSRs) were conducted using CandiSSR pipeline (Xia et al., 2016), with default parameters. The data visualization was also accomplished using the OmicStudio tools (Lyu et al., 2023).

### 2.6 Identification of single-copy nuclear genes

To retrieve Angiosperms353 target genes (Johnson et al., 2019) within the genome of *D. nipponica*, low-coverage whole genome sequencing data from each *D. nipponica* accession were independently subjected to HybPiper v. 2.1.6 (Johnson et al., 2016) for assembling sequences for each gene, with all settings at default. Briefly, paired-end clean reads from each accession were mapped to target genes with bwa v. 0.7.17 (Li and Durbin, 2009). Subsequently, mapped reads were organized into distinct directories and assembled into contigs using SPAdes v.3.13.1 (Bankevich et al., 2012). These assembly contigs were then aligned to their associated target sequences using Exonerate v.2.2 (Slater and Birney, 2005). Finally, the recovered gene sequences were extracted using the HybPiper script called retrieve_sequences.py. Additionally, recovery statistics were generated using the two Python scripts, namely get_seq_lengths.py and hybpiper_stats.py, which are included in the HybPiper pipeline (Johnson et al., 2016). The resulting gene sequences shared across all eight *D. nipponica* accessions were imported into Geneious Prime® 2022.0.1, and aligned individually with the multiple alignment plugin MAFFT v.7 (Katoh and Standley, 2013). After excluding those alignments with pairwise identity below 90%, the remaining alignments were concatenated into a supermatrix for nuclear phylogenetic analyses.

### 2.7 Phylogenetic analyses within *D. nipponica*

For plastome phylogenetic analyses, maximum likelihood (ML) and Bayesian inference (BI) analyses were conducted based on two distinct datasets: complete plastome sequences and a set of 80 shared protein coding regions present in all eight accessions examined in this study (**Table 1**), with *D. zingiberensis* (OQ526000) as an outgroup. Both complete plastome sequences and protein coding sequences were aligned using MAFFT v.7 plugin (Katoh and Standley, 2013) in Geneious Prime® 2022.0.1. The best-fitting substitution model, GTR + I + G, for each dataset was determined based on the Akaike Information Criterion (AIC) as computed by jModelTest v.2.1.4 (Darriba et al., 2012). The ML analyses were performed using RAxML v.8.2.12 (Stamatakis, 2014) in the CIPRES Science Gateway v.3.3 (http://www.phylo.org/portal2/), with 1000 bootstrap replications. The BI analyses were conducted using MrBayes v.3.2.7 (Ronquist et al., 2012), comprising two independent runs of 1 × 10^6^ generations. Each run employed four independent Markov chain Monte Carlo (MCMC) chains, consisting of one cold chain and three heated chains, with a sampling frequency of 1000 trees. The first 1000 trees were discarded as ‘burn-in’, and the remaining trees were used to construct a majority-rule consensus tree and estimate the posterior probabilities (PPs).

For nuclear phylogenetic analyses, clean reads of *D. zingiberensis* (Cheng et al., 2021) were mapped to the 17 single-copy nuclear genes (17-SCNGs) shared by the eight *D. nipponica* accessions (as detailed in the Results) to yield the 17-SCNGs of *D. zingiberensis*, through CLC Genomics Workbench v.23.0.4 (https://digitalinsights.qiagen.com). Subsequently, the 17-SCNGs shared by the eight *D. nipponica* accessions and *D. zingiberensis*, which served as the outgroup, were aligned using MAFFT v.7 plugin (Katoh and Standley, 2013) in Geneious Prime® 2022.0.1. Both ML and BI analyses were implemented following the same methods described as above for plastome sequences.

## 3 Results

### 3.1 Plastome features of *D. nipponica*

**Figure 1:**
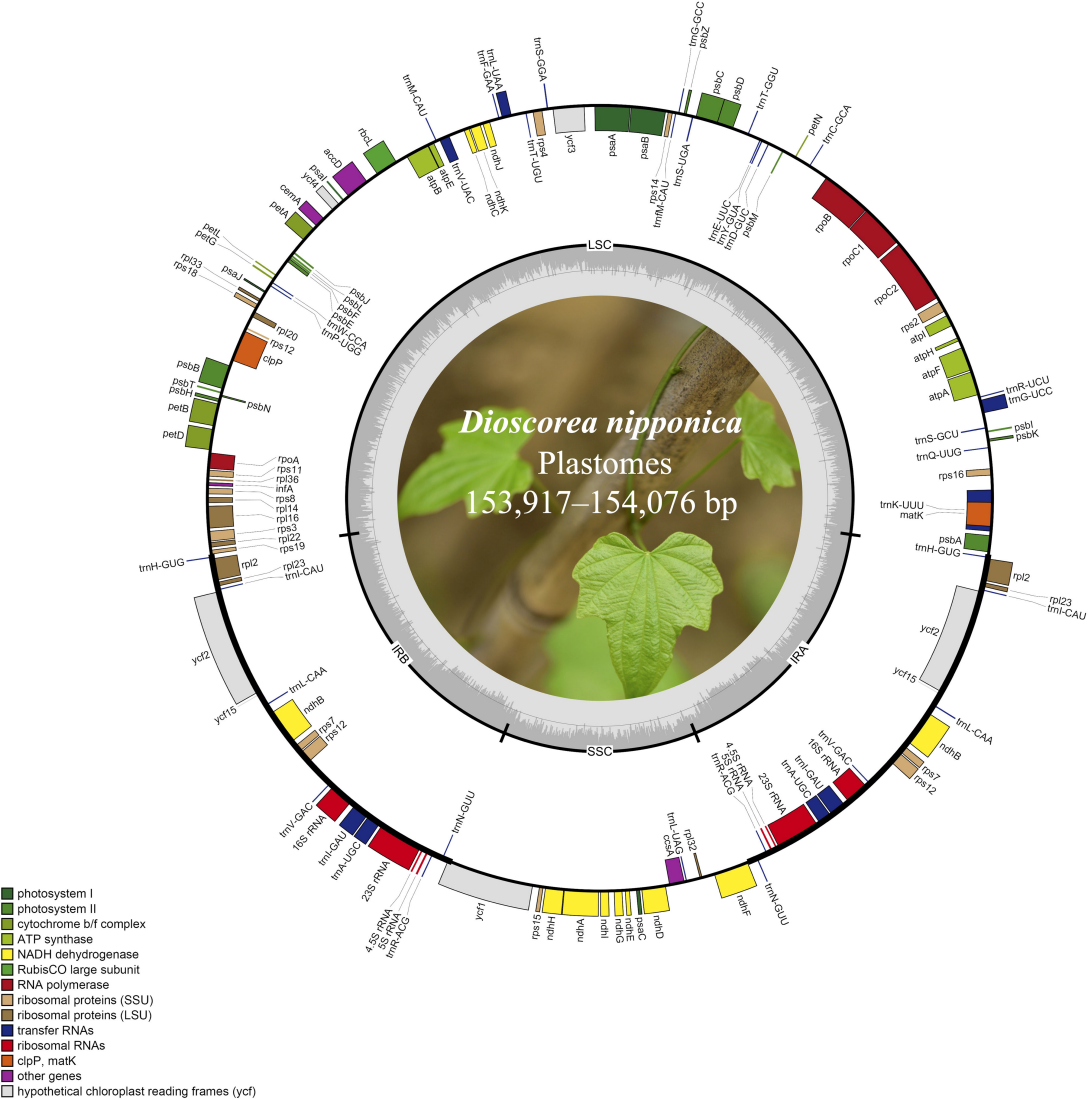
The plastome maps of *D. nipponica* accessions. Genes shown on the outside of the circle are transcribed clockwise, and those inside counter-clockwise. Genes associated with different functional categories are color coded. The darker grey in the inner ring corresponds to the GC content and the lighter grey to the AT content. The *D. nipponica* plant was displayed within the inner circle.

Illumina paired-end (150 bp) sequencing produced 23,737,170–33,758,516 clean reads for these eight *D. nipponica* accessions (**Table 1**). The coverage depths resulting from mapping the Illumina reads to the plastome sequences ranged from 451× (JN) to 2631× (BJ) (**Table 1**). Additionally, both GetOrganelle and NOVOPlasty generated identical plastome assemblies. Taken together, these results indicated a high-quality and accuracy of our plastome assemblies. The whole plastome sequences of the eight *D. nipponica* accessions exhibited a narrow range in size, spanning from 153,917 bp (BJ and HeN) to 154,076 bp (FD) (**Figure 1**; **Table 1**). The plastome of *D. nipponica* maintained the typical circular quadripartite structure, consisting of a pair of inverted repeat (IR) regions (25,508–25,530 bp) separated by a large single copy (LSC) region (83,982–84,108 bp), and a small single copy (SSC) region (18,908–18,919 bp) (**Table 1**). Notably, the lengths of the IR regions in six Chinese accessions were consistent at 25,508 bp, representing a 22 bp reduction when compared to the IR regions in the two Japanese accessions measuring 25,530 bp each (**Table 1**). The GC content of whole plastome sequences (37.20%), LSC (35.00%) and IR (43.00%) regions were identical across the eight *D. nipponica* accessions, while in the SSC region, the GC content in six Chinese accessions (31.20%) was slightly lower than that in two Japanese accessions (31.30%) (**Table 1**).

The plastomes of all eight *D. nipponica* accessions exhibited remarkable uniformity in terms of gene count, content, and arrangement. They shared an identical set of 114 unique genes, comprising 80 protein-coding genes (PCGs), 30 transfer RNA (tRNA) genes, and four ribosomal RNA (rRNA) genes. Among these, nineteen unique genes (comprising seven PCGs, eight tRNA genes, and all four rRNAs) were duplicated within the IRs, resulting in a total gene count of 133 (**Figure 1**; **Table S1**). Within this set of unique genes, nine PCGs (namely *atpF*, *ndhA*, *ndhB*, *petB*, *petD*, *rpl2*, *rpl16*, *rpoC1*, and *rps16*) and six tRNAs (*trnA*-*UGC*, *trnG*-*UCC*, *trnI*-*GAU*, *trnK*-*UUU*, *trnL*-*UAA*, and *trnV*-*UAC*) each contained a single intron, while three PCGs (*clpP*, *rps12*, and *ycf3*) harbored two introns (**Figure 1**; **Table S1**).

### 3.2 Plastome-derived SSRs

**Figure 2:**
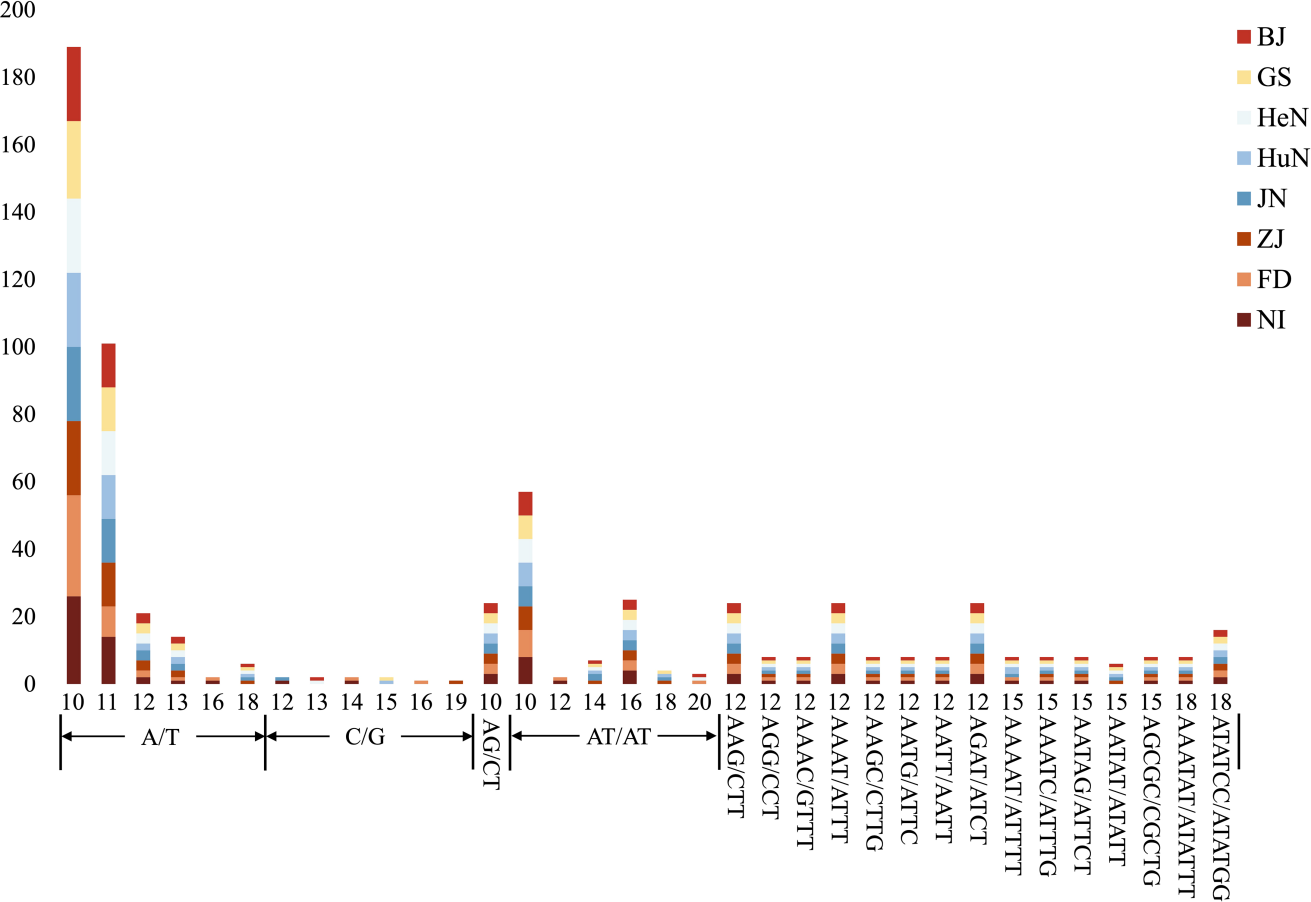
The lengths and motif types of plastome-derived SSRs in eight *D. nipponica* accessions.

The MISA analysis detected a total of 639 simple sequence repeats (SSRs) derived from the plastomes of the eight different *D. nipponica* accessions. The number of plastome-derived SSRs for each accession ranged from 78 (for ZJ) to 83 (for NI). It appeared that the two Japanese accessions (with 82–83 SSRs) contained more SSRs compared to the six Chinese accessions (with 78–80 SSRs) (**Figure 2**; **Table S2**). Among these plastome-derived SSRs, mononucleotide repeats were the most prevalent, varying from 41 (for HuN) to 46 (for NI), followed by dinucleotide repeats (15–16 for each accession) and tetranucleotide repeats (10 for each accession). In contrast, trinucleotide repeats (4 for each accession), pentanucleotide repeats (ranging from 4 to 6 for each accession), and hexanucleotide repeats (3 for each accession) were relatively less common in the *D. nipponica* accessions (**Figure 2**; **Table S2**). The most frequent motifs observed were A/T and AT/TA for mono- and dinucleotide repeats, constituting 50.63%–53.01% and 15.00%–15.85% of the total plastome-derived SSRs across all eight *D. nipponica* accessions, respectively. Additionally, a set of at least four plastome-derived SSRs, namely (A/T)_16_, (A/T)_18_, (C/G)_14_, and (AATAT/ATATT)_3_, could effectively distinguish between the Chinese and Japanese groups among the eight *D. nipponica* accessions (**Figure 2**; **Table S2**).

### 3.3 Plastome-divergent hotspots

**Figure 3:**
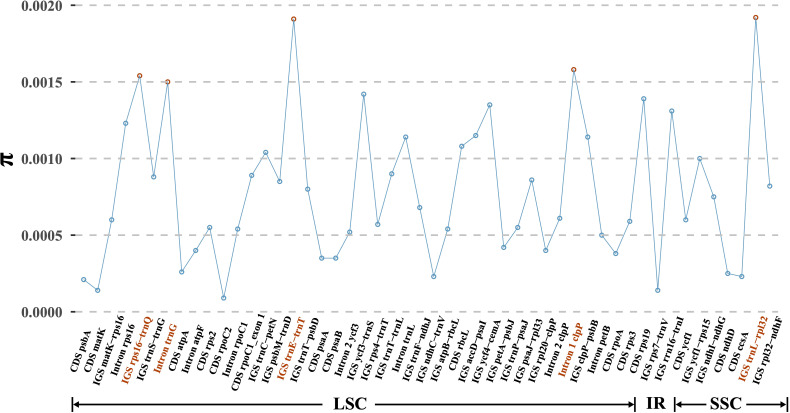
Nucleotide variability (*π*) values of 50 regions (15 CDS, 26 IGS, and 9 introns) extracted from the alignment matrix of plastome sequences encompassing eight *D. nipponica* accessions. Three IGS regions (*trnL*-*rpl32*, *trnE*-*trnT*, and *rps16*-*trnQ*), along with two introns (Intron 1 of *clpP* and Intron of *trnG*), exhibiting π values exceeding 0.15%, were highlighted in red.

Based on the multiple alignment of eight *D. nipponica* plastomes, a total of 50 regions, comprising 15 coding regions (CDS), 26 intergenic spacers (IGS), and 9 intron regions, were isolated for the purpose of computing nucleotide diversity (*π*). The *π* values ranged from 0.01% (CDS *rpoC2*) to 0.19% (IGS *trnL*-*rpl32*) (**Figure 3**). Among these regions, CDS *ycf1* stood out as the most diverse coding sequence, while the top three intergenic spacers (*trnL*-*rpl32*, *trnE*-*trnT*, and *rps16*-*trnQ*) and two intron regions (Intron 1 of *clpP* and Intron *trnG*) exhibited particularly high levels of variability, each surpassing 0.15% (**Figure 3**). These six regions hold significant promise for assessing intraspecific genetic variability of *D. nipponica*.

**Figure 4:**
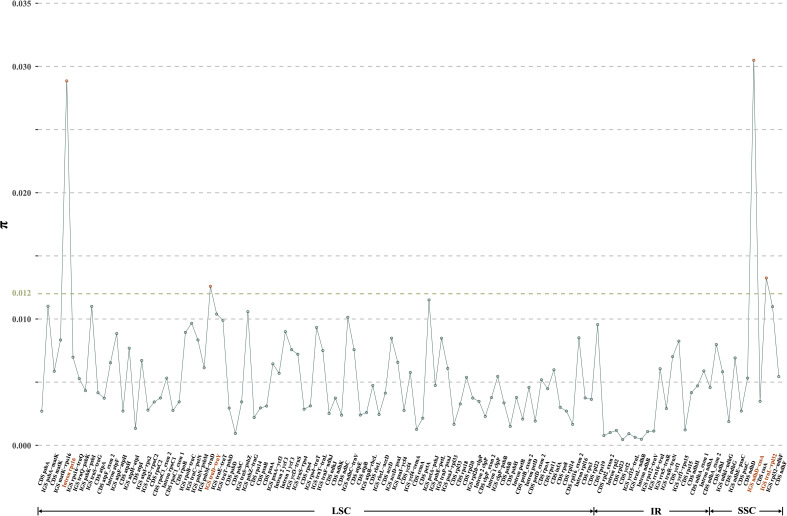
Nucleotide variability (*π*) values of 119 regions (57 CDS, 49 IGS, and 13 introns) extracted from the alignment matrix of plastome sequences encompassing *D. nipponica* and its closely related species, i.e., *D. collettii*, *D. gracillima*, *D. villosa*, and *D. zingiberensis*. Three IGS regions (i.e., *ndhD*-*cssA*, *trnL*-*rpl32*, *trnD*-*trnY*), and the intron *rps16* exhibiting π values exceeding 1.20%, were highlighted in red.

Moving to the inter-specific level, a total of 119 regions (57 CDS, 49 IGS, and 13 introns) were eventually isolated for the calculation of nucleotide diversity, yielding *π* values ranging from 0.05% (CDS *ycf2*) to 3.05% (IGS *ndhD*-*cssA*) (**Figure 4**). Significantly, three IGS regions (i.e., *ndhD*-*cssA*, *trnL*-*rpl32*, and *trnD*-*trnY*), and the intron *rps16* emerged as the four most highly variable regions (with *π* values exceeding 1.20%) (**Figure 4**), which could be used for distinguishing *D. nipponica* from its closely related species.

### 3.4 Polymorphic nuclear SSRs

**Figure 5:**
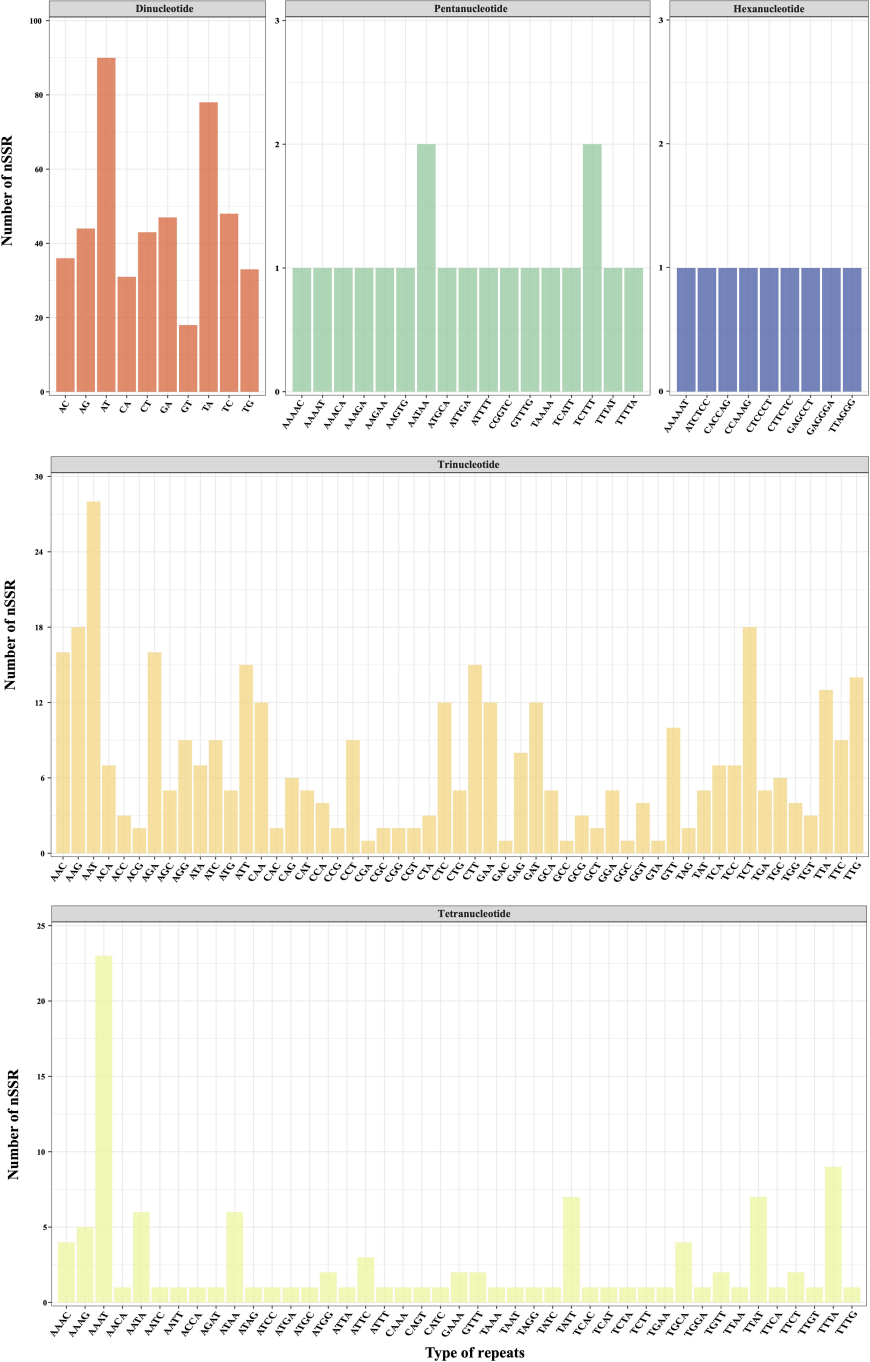
Polymorphic nuclear SSRs across eight *D. nipponica* accessions.

Utilizing low-coverage whole-genome sequencing data, we generated a range of 1,262,131–1,685,719 scaffolds for each *D. nipponica* accession, with N50 Length varying between 285–317 bp (**Table S3**). All these scaffolds have been deposited at Dryad under the existing accession number (https://doi.org/10.5061/dryad.05qfttf96). Based on these scaffolds, a total of 2417 candidate polymorphic nuclear SSRs (PolynSSRs) were initially generated for *D. nipponica*. After filtering out those low-quality PolynSSRs with a missing rate (MR) ≥ 0.5 and transferability (similarity) < 95%, a subset of 988 high-quality candidate PolynSSRs were remained (**Figure 5**; **Table S4**). Within this group of high-quality candidate PolynSSRs, it was possible to design primers for 980 of them, constituting 99.19% of the total (**Table S4**). Notably, among 988 high-quality candidate PolynSSRs, dinucleotide repeats (468, 47.37%) were the most abundant, followed by trinucleotide (380, 38.46%) and tetranucleotide (112, 11.34%) repeats (**Figure 5**). In contrast, pentanucleotide repeats (19, 1.92%) and hexanucleotide repeats (9, 0.91%) were the least frequently encountered types (**Figure 5**; **Table S4**).

### 3.5 Single-copy nuclear genes

Clean reads from these eight accessions were collectively mapped to 351–352 genes, yielding 37 to 117 sequences for each respective accession (**Table 2**). Notably, among these sequences, 18 were found to be shared across all eight accessions. Following the exclusion of one alignment with pairwise identity below 90%, a supermatrix with a total length of 2154 bp was formed by concatenating the remaining 17 gene sequences. This supermatrix was subsequently utilized for nuclear phylogenetic analyses. However, it’s worth emphasizing that only a handful of sequences (6 for BJ, 19 for GS, 3 for HeN, 2 for HuN, 25 for JN, 27 for ZJ, 28 for FD, and 18 for NI, respectively), each with a length comprising at least 50% of the target, were successfully recovered (**Table 2**).

**Table 2:** The recovery efficiency of Angiosperms353 target genes in eight *D. nipponica* accessions.

Accession	NumReads	ReadsMapped	GenesMapped	GenesWithSeq	GenesAt25pct	GenesAt50pct
BJ	30324374	445246	352	47	14	6
GS	31352778	409836	352	94	44	19
HeN	23737170	325236	352	39	6	3
HuN	24959536	332371	352	37	7	2
JN	32495028	449107	352	116	48	25
ZJ	31790950	506942	352	103	34	27
FD	33758516	540743	352	117	49	28
NI	28825392	482733	351	96	42	18

NumReads: total number of input reads in the *.fastq files provided; ReadsMapped: total number of input reads that mapped to sequences in the target file; GenesMapped: Number of genes in the target file that had reads mapped to their representative sequences; GenesWithSeq: Number of genes with sequences; GenesAt25pct and GenesAt50pct: Number of genes with sequences > 25% and 50% of the mean target length, respectively.

### 3.6 Intraspecific phylogenetic relationship of *D. nipponica*

**Figure 6:**
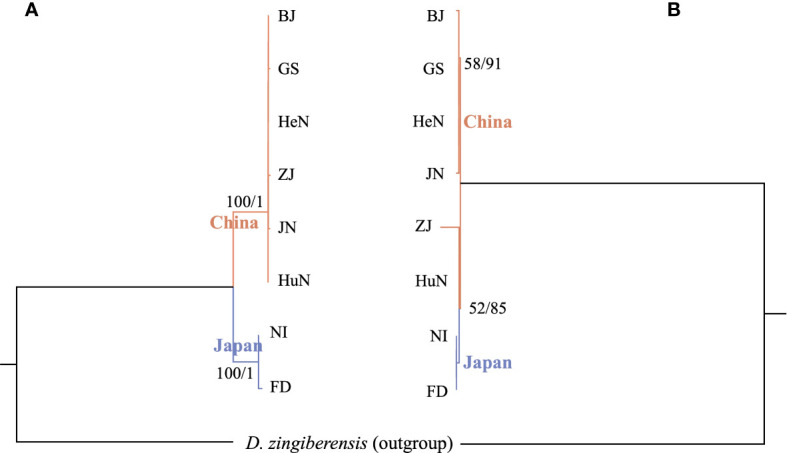
Intraspecific phylogenetic trees of *D nipponica* inferred from **(A)** complete plastome sequences and 80 shared protein coding regions, as well as **(B)** 17 single copy nuclear genes, based on the methods of maximum likelihood (ML) and Bayesian inference (BI). The ML bootstrap values/BI posterior probabilities were displayed above the lines.

Both the ML and BI analyses, conducted using complete plastome sequences and 80 shared protein coding regions, provided robust support for the division of *D. nipponica* into distinct Chinese and Japanese groups (**Figure 6A**), with high bootstrap values (BS values = 100%) and posterior probabilities (PPs = 1.0). Notably, no evident genetic distinction was observed within the Chinese group (**Figure 6A**). However, when phylogenetic analyses were conducted based on 17 single-copy nuclear genes, they indicated that the two Japanese accessions formed a monophyletic group with two Chinese accessions (HuN and ZJ) from Central and South China. These, in turn, constituted a sister group with the other four Chinese accessions (BJ, GS, JN, and HeN) from North China (**Figure 6B**).

## 4 Discussion

### 4.1 Plastome characteristics and plastome-derived markers of *D. nipponica*

Plastomes have become a cornerstone in the realm of plant phylogenetics and evolutionary studies, primarily due to their unwavering preservation of gene order and the striking absence of heteroplasmy and recombination (Birky et al., 1983; Daniell et al., 2016). Notably, plastomes exhibit uniparental inheritance, typically maternal in angiosperms and paternal in gymnosperms, offering a distinctive avenue for unraveling the respective contributions of seed and pollen dispersal to the genetic makeup of natural populations—a perspective enriched when contrasted with nuclear markers (Birky, 1995; Mohammad-Panah et al., 2017). In this study, the investigation of plastome features in eight *D. nipponica* accessions unveiled a striking uniformity in terms of gene count, content, and arrangement (**Figure 1**; **Table 1**), which suggested an enduring evolutionary stability within *D. nipponica*.

The differences in the sizes of inverted repeats (IRs) and the variations observed at four distinct junctions (IRa/SSC, IRa/LSC, IRb/SSC, and IRb/LSC) often significantly influence the overall size of the plastomes and the count of genes, across angiosperm species (Sun et al., 2016). Remarkably, within a single species, the lengths of IR regions were always consistently identical among different individuals or accessions (e.g., *Zizania latifolia*, Lu et al., 2022; *Dioscorea alata*, Lu et al., 2023), playing a crucial role in stabilizing plastomes (Blazier et al., 2016). Our study, in contrast to earlier findings indicating uniform IR length within a specific species, revealed discrepancies in the length of IR regions between Chinese and Japanese accessions (**Table 1**). This discovery may signify historical divergence or unique evolutionary history of *D. nipponica* accessions in these distinct geographic regions (Jansen and Ruhlman, 2012).

Recent studies have underscored the role of simple sequence repeats (SSRs) in adaptation, survival, and evolution of species (Labbé et al., 2011; Yuan et al., 2021). The differences observed in plastome-derived SSRs between Chinese and Japanese accessions (**Figure 2**), hold significance in understanding the genetic variations and evolutionary history of *D. nipponica*. It is plausible that geographic isolation, differing environmental factors, and historical events have led to genetic differences between these distinct geographical accessions (Qiu et al., 2011). Further exploration of these variations and their correlation with historical events and/or environmental factors, particularly within the context of a population genetic framework, could provide valuable insights into the evolutionary history and population dynamics of *D. nipponica*.

Due to their smaller effective population sizes compared to nuclear genomes, as well as the limited gene dispersal via seeds as opposed to pollen-mediated gene flow, plastome-derived markers have the potential to serve as effective indicators for historical bottlenecks, founder effects, and genetic drift (Mohammad-Panah et al., 2017). Regrettably, when this study was initiated, plastome-derived markers tailored for *D. nipponica* were very limited. The present study successfully identified a significant array of plastome-derived SSRs, ranging from 78 for ZJ to 83 for NI (**Figure 2**; **Table S2**), as well as six mutational hotspots (CDS *ycf1*, *trnL*-*rpl32*, *trnE*-*trnT*, *rps16*-*trnQ*, Intron 1 of *clpP*, and Intron *trnG*) (**Figure 3**). These newly discovered plastome-derived markers hold substantial promise as valuable tools for population genetics investigations and phylogenetic analyses of *D. nipponica*.

### 4.2 Nuclear genomic resources for *D. nipponica*

Nuclear SSR markers (nSSRs) have proven to be invaluable tools in various aspects, such as population genetic analyses, identification of germplasm resources, and marker-assisted breeding programs, due to their co-dominant inheritance, adherence to Mendelian inheritance principles, wide genomic distribution, high polymorphism, and verifiable neutrality (Kaldate et al., 2017; Lu et al., 2022). In this study, we made a noteworthy discovery of 988 high-quality candidate polymorphic nSSRs (**Figure 5**; **Table S4**), which hold immense potential in shedding light on the genetic diversity and population structure of *D. nipponica*. More importantly, conducting comparative analyses of nSSRs and plastome-derived SSRs may provide complementary and sometimes contrasting perspectives on the genetic structure, differentiation, and gene flow (pollen- and seed-mediated) among *D. nipponica* populations (Mohammad-Panah et al., 2017). Among these molecular markers, five polymorphic nSSRs (i.e., nSSR_901, nSSR_1065, nSSR_1163, nSSR_1491 and nSSR_2102) showing differentiation between BJ and FD, along with five plastome-derived SSRs were selected for validation using PCR-based Sanger sequencing (**Supplementary Data**). The sequence validation showed 100% similarity, affirming the accuracy and reliability of these identified molecular markers. Undoubtedly, the genomic resources described here formed a foundational platform for future studies in population genetics, evolution, and breeding of *D. nipponica*, crucial for developing effective conservation strategies, understanding the genetic basis of adaptation, and designing suitable breeding programs.

Single-copy nuclear genes (SCNGs), characterized by bi-parental inheritance, higher evolutionary rates, and numerous unlinked loci (Alvarez et al., 2008; Hojjati et al., 2019), offer significant potential for addressing issues related to hybridization and incomplete lineage sorting, potentially reconciling discrepancies among plastome genes (Small et al., 2004; Doyle, 2022). However, the recovery of SCNGs in this study was constrained (**Table 2**), possibly due to low sequencing coverage. Our previous flow cytometry analysis indicated genome sizes of ~550 Mb and ~1.10 Gb for diploids (2n = 20) and tetraploids (2n = 40), respectively (detailed data not shown), thus the total clean data generated in this study (**Table 1**) represented about 8× and 4× coverage of the estimated diploid and tetraploid genomes, respectively. Although the sequencing coverage was somewhat lower than optimal minimum sequencing depth (10×) for high-quality SCNG assembly via low-coverage genome sequencing (Liu et al., 2021), the successful retrieval of 17 SCNGs from diverse *D. nipponica* accessions underscored their potential in resolving phylogenetic relationships within this species. Efforts aimed at obtaining more comprehensive SCNG sequences, possibly through improved sequencing methodologies or increased coverage, will be imperative. Addressing these limitations will undoubtedly enhance the accuracy and robustness of phylogenetic reconstructions, providing a more nuanced understanding of the intraspecific phylogenetic relationships of *D. nipponica*.

### 4.3 Intraspecific phylogenetic relationship of *D. nipponica*

The taxonomic classification and intraspecific phylogenetic relationships within *D. nipponica* have long been the subject of ongoing debate and controversy (Ding and Gilbert, 2000; Gao et al., 2008; Li et al., 2020; Hu et al., 2023). However, previous taxonomic and phylogenetic studies concerning *D. nipponica* have often grappled with limitations, either relying on short DNA sequences or featuring a restricted sample of taxa, resulting in constrained and sometimes conflicting conclusions (Gao et al., 2008; Li et al., 2020; Hu et al., 2023). Although in this study, phylogenetic analyses based on plastome sequences and concatenated SCNG data, revealed cytonuclear discordance within *D. nipponica*: the former supported the differentiation of *D. nipponica* accessions into distinct Chinese and Japanese groups (**Figure 6A**), while the latter suggested that the Japanese accessions formed a monophyletic group with two Chinese accessions (HuN and ZJ) from Central and South China (**Figure 6B**), both findings challenged the current subspecies classification as per the Flora of China (Ding and Gilbert, 2000) and the proposition to elevation of *D. nipponica* subsp. *rosthornii* to the status of an independent species (Gao et al., 2008; Li et al., 2020). This cytonuclear discordance may be attributed to both ancient and recent hybridization events as well as polyploidization occurrences within this species (Ding and Gilbert, 2000). Alternatively, it could be caused by the lack of phylogenetic resolution in the SCNG data. The nuclear gene-based phylogeny is inadequate in offering definitive insights into intraspecies relationships within *D. nipponica* (see **Figure 6B**), highlighting the inherent challenges of resolving deeper phylogenetic relationships with a limited set of nuclear genes. Thus, to gain a more comprehensive understanding of the taxonomy and evolutionary history of *D. nipponica*, it is imperative to utilize more robust molecular markers, such as nuclear SNPs, and conduct broader sampling across various regions.

## 5 Conclusions

In this study, we conducted low-coverage whole genome sequencing of diverse *D. nipponica* accessions, to retrieve plastome information, including whole plastome sequences, plastome-derived SSRs and plastome-divergent hotspots, as well as nuclear genomic markers, including polymorphic nuclear SSRs and single-copy nuclear genes. Our findings revealed a striking uniformity in plastome features across diverse *D. nipponica* accessions, with subtle length differences in inverted repeat regions between Chinese and Japanese accessions. A total of 639 plastome-derived SSRs and six divergent hotspots were identified from *D. nipponica* plastomes. Besides, four highly divergent hotspots were developed as potential markers for distinguishing *D. nipponica* from its closely related species. In parallel, 988 high-quality candidate polymorphic nuclear SSRs and 17 single-copy nuclear genes were obtained. The genomic resources identified here will aid in the conservation and strategic utilization of this economically significant plant. Furthermore, the study shed light on the intraspecific phylogenetic relationships of *D. nipponica*, challenging the current subspecies classification and highlighting the need for further taxon sampling and the integration of more robust molecular markers to thoroughly unravel the intraspecific relationship and evolutionary history of this species.

## Data availability statement

The datasets presented in this study can be found in online repositories. The names of the repository/repositories and accession number(s) can be found in the article/**Supplementary Material**.

## Author contributions

KH: Software, Writing – original draft. MC: Software, Writing – original draft. PL: Resources, Writing – review & editing. XS: Resources, Writing – review & editing. RL: Conceptualization, Resources, Writing – review & editing.
