# Supplementary material for: Intraspecific phylogeny and genomic resources development for an important medical plant Dioscorea nipponica, based on low-coverage whole genome sequencing data
Source: Front Plant Sci. 2023 Dec 12;14:1320473. doi: 10.3389/fpls.2023.1320473 (PMC10749966; doi:10.3389/fpls.2023.1320473)
Supplement: Supplementary file 3 [file Table_3.docx]

**Table S3** Summary of nuclear genome assemblies of all eight *Dioscorea nipponica* accessions

| Accession | Total number of Scaffolds | Total Length (bp) | Minimum length (bp) | Maximum length (bp) | N50 (bp) | Mean (bp) |
| --- | --- | --- | --- | --- | --- | --- |
| BJ | 1,401,564 | 443,891,469 | 100 | 9543 | 443 | 317 |
| GS | 1,437,798 | 435,585,419 | 100 | 8082 | 402 | 303 |
| HeN | 1,263,288 | 397,976,070 | 100 | 6862 | 422 | 315 |
| HuN | 1,262,131 | 384,601,179 | 100 | 7059 | 396 | 305 |
| JN | 1,385,776 | 436,950,176 | 100 | 9612 | 440 | 315 |
| ZJ | 1,484,200 | 441,333,045 | 100 | 7704 | 390 | 297 |
| FD | 1,685,719 | 480,726,826 | 100 | 8152 | 351 | 285 |
| NI | 1,434,592 | 423,909,274 | 100 | 5814 | 363 | 296 |
