# Supplementary material for: Intraspecific phylogeny and genomic resources development for an important medical plant Dioscorea nipponica, based on low-coverage whole genome sequencing data
Source: Front Plant Sci. 2023 Dec 12;14:1320473. doi: 10.3389/fpls.2023.1320473 (PMC10749966; doi:10.3389/fpls.2023.1320473)
Supplement: Supplementary file 5 [file Table_5.docx]

**Plastome-derived SSR**

**(A/T)_18_ for BJ, (A/T)_16_ for FD**

**BJ:**

TATTCTGATATTTAAATTCGATATACATTTTTTTTGTAAAAGCGGATTTTTTGATTTCCTTGGACCACGCAAAAATTTGTTAATATTTCCGATTTCATCTTCTTGTTACCGGATGCTCCATAGGAATAAATCATTATTCTTTTCCACTACAGATAAATATTTCAAAAATCTATTTTTTAATATTTTTATTTGATTCCAAAATTGGATATTGTTTTGTTGTTGCAACAATACAATAGAGAATAAATGTGAGAAGGGAAAGGGGCTTTCATTTCCAGTCTACTATATATTTAATATTTAATTGGGGGTCAGGGAAAAATAGGGAATTTTCTTTTTTTTTTTTTTTTTTACCCATTAATATACTCGGTGGTATGAGTCATAGGGCAATTAATTCGGGGTTCAAATGGTTATTAAGAATGGGAATAGTAAGGGCTAATCGAATTGAACTGATGGATTTACCTAGGTTGGTTTATGGTCCAATAGAAAAGAAGAATTTGTATCTTCGAAACCTATCGTAAAAGGCATTGAACGAGGATCGAACTATTGTATGCCCCCCAAATGATATGAGGTGTTCGGAAATGGTTGAAGTAGTTGAATAGGAGGATCACTATGACTATAGCCGCAGGTAGATTTACCAAAGAAGAA

**FD:**

TATTCTGATATTTAAATTCGATATACATTTTTTTTGTAAAAGCGGATTTTTTGATTTCCTTGGACCACGCAAAAATTTGTTAATATTTCCGATTTCATCTTCTTGTTACCGGATGCTCCATAGGAATAAATCATTATTCTTTTCCACTACAGATAAATATTTCAAAAATCTATTTTTTAATATTTTTATTTGATTCCAAAATTGGATATTGTTTTGTTGTTGCAACAATACAATAGAGAATAAATGTGAGAAGGGAAAGGGGCTTTCATTTCCAGTCTACTATATATTTAATATTTAATTGGGGGTCAGGGAAAAATAGGGAATTTTCTTTTTTTTTTTTTTTTGCCCATTAATATACTCGGTGGTATGAGTCATAGGGCAATTAATTCGGGGTTCAAATGGTTATTAAGAATGGGAATAGTAAGGGCTAATCGAATCGAACTGATGGATTTACCTAGGTTGGTTTATGGTCCAATAGAAAAGAAGAATTTGTATCTTCGAAACCTATCGTAAAAGGCATTGAACGAGGATCGAACTATTGTATGCCCCCCAAATGATATGAGGTGTTCGGAAATGGTTGAAGTAGTTGAATAGGAGGATCACTATGACTATAGCCGCAGGTAGATTTACCAAAGAAGAA

**(C/G)_13_ for BJ, (C/G)_16_ for FD**

**BJ:**

ACCACCAAAATGGGTGGATCCGATTAAATTATAAAGTAAAAAACAACAAAGAAATTCCTAACAAATTATATTCCTAATATAGATGTCAAAATTGACAGACTTATCCATTTTTTTTTTTATTTTCGTTACTAAAATACGCCTTGTCTTCTATAATTTAATTATAGGGTAAATCCGTCAAACCATCATTTGGATGAACTGAAGGAAAACCCAATCAATATAGGGTGGGTGTAAACAGATCCATTTATTTATGATCGAATTATATTTGTTCAATACACCATTGTCAATATCAATGGAATGTTGAGAAAACAAATCAAATTATAAGTAAATCAAATAATGACTTGTGTTTGGACTTAAATAATGAATTTGATGGGAAAAAATAAAGAATAGGAAGAAAAAAATCTCGGATTTATTCAATCAATAGAGATACAATAAACAAGATTAATCTCCTGTTTTGGATTCCACTAACAAAAGAAAAATAAAAATATGAATAGAGCAAAAAAAGGTAAATAGTGAGGAAATAATTACACTAATTAAACAAAGATAAATACTAGTTACCCCCCCCCCCCCTTTTTTTTTATTCATTAATTTAGTTTTCCTTTAATTTTGAATTTTCATTTTTAATTAATTTAACTGGAAATTCTTTCTTTAAAAAATTCTTTTTTTTTTAACTTTGCCTTGCTTAAAATCTCATGAACAGTTCCTATAATAGGTTAAATGCCTTTTTCAAGGAAATATAGAATAGTGGGAACATTTAAATAAGTTTGATTATTCATCGGATCGTAAAAACCCACTTTTTGAAGATCTCTTGCTTCTCTTGGGAATCGAACATCAATTGCAACG

**FD:**

ACCACCAAAATGGGTGGATCCGATTAAATTATAAAGTAAAAAACAACAAAGAAATTCCTAACAAATTATATTCCTAATATAGATGTCAAAATTGACAGACTTATCCATTTTTTTTTTTATTTTCGTTACTAAAATACGCCTTGTCTTCTATAATTTAATTATAGGGTAAATCCGTCAAACCATCATTTGGATGAACTGAAGGAAAACCCAATCAATATAGGGTGGGTGTAAACAGATCCATTTATTTATGATCGAATTATATTTGTTCAATACACCATTGTCAATATCAATGGAATGTTGAGAAAACAAATCAAATTATAAGTAAATCAAATAATGACTTGTGTTTGGACTTAAATAATGAATTTGATGGGAAAAAATAAAGAATAGGAAGAAAAAAATCTCGGATTTATTCAATCAATAGAGATACAATAAACAAGATTAATCTCCTGTTTTGGATTCCACTAACAAAAGAAAAATAAAAATATGAATAGAGCAAAAAAAGGTAAATAGTGAGGAAATAATTACACTAATTAAACAAAGATAAATACTAGTTACCCCCCCCCCCCCCCCTTTTTTTTTTTATTCATTAATTTAGTTTTCCTTTAATTTTGAATTTTCATTTTTAATTAATTTAACTGGAAATTCTTTCTTTAAAAAATTCTTTTTTTTTTAACTTTGCCTTGCTTAAAATCTCATGAACAGTTCCTATAATAGGTTAAATGCCTTTTTCAAGGAAATATAGAATAGTGGGAACATTTAAATAAGTTTGATTATTCATCGGATCGTAAAAACCCACTTTTTGAAGATCTCTTGCTTCTCTTGGGAATCGAACATCAATTGCAACG

**(TCC)_4_ for BJ and FD, respectively**

**BJ:**

CTAAATTAAGACTGAACTAAAAGATAAACAAAGTAAAGCTAAATCTATTGAAGTACTACAAACTTTGAAGTAGTACAAACAAAGTAGAATGGAATAAATTGTATCAATATCCGGATTTTTTGTATATGTGTAAAATGTATATATAAGAAATTGAGACTCTGTCTTCTGATTTGTTTTATAGAAATCTATCTAATTCCTCCTCCTCCAATTAATTTTGTATTTGTAGTTACAAGTTATCACGACATAATAGATAGGATTGGCGACCCAACATTTGGAGAAAAGGAGAGGGTAGATTATCAATCATGGAAAAAATAGATAGAGAAAA

**FD:**

CTAAATTAAGACTGAACTAAAAGATAAACAAAGTAAAGCTAAATCTATTGAAGTACTACAAACTTTGAAGTAGTACAAACAAAGTAGAATGGAATAAATTGTATCAATATCCGGATTTTTTGTATATGTGTAAAATGTATATATAAGAAATTGAGACTCTGTCTTCTGATTTGTTTTATAGAAATCTATCTAATTCCTCCTCCTCCAATTAATTTTGTATTTGTAGTTACAAGTTATCACGACATAATAGATAGGATTGGCGACCCAACATTTGGAGAAAAGGAGAGGGTAGATTATCAATCATGGAAAAAATAGATAGAGAAAA

**(TTC)_4_ for BJ and FD, respectively**

**BJ:**

CGTTCCTCTTTTTTAGTTTAGTTCTTAGGATAGGAAAGAAAAAGTGTATTGAACCTCAGAAAAATGCAGTGGATCTAAGATTGAATTTTATTGAATTTTGGAGAACAGAAATAGAAATGTGAGTCCAATATATTCTATGGGCAGGCTCCACGAAATCAAATCAAATCATAAGAGAAAAAAGATACTTAAATGAAATACCGAGATTAGGGTAGTAGGATGCTGGTTCGAAAGTAATTGTATTACTTAATTATTACTCAATTAATATTAATTTAATTACAACCAAATCTTTAGAAATTCCATTTCTAGTTAGTAACTTCTATTGATTTTTTTGTTCTTTTCTTCTTCTTCGGTTCGGATCGAAAATAGAAGAATTTAAGTCGATCAAAAGGAGGTTCATGGCCAAGGGTAAGGATGTCAGAGTCAGAGTTATTTTGGAATGCGCCAGTTGTGTCCGAAATGGTATCAATAAAGAATTGCCGGGTATTTCTAGATATATTACTCAAAAGAGTCGACACAATACACCCAATCGATTAGAATTAAGAAAATTTTGTCGCTATTGTCGCACACATACGATTCATGGAGAAATAAAGA

**FD:**

CGTTCCTCTTTTTTAGTTTAGTTCTTAGGATAGGAAAGAAAAAGTGTATTGAACCTCAGAAAAATGCAGTGGATCTAAGATTGAATTTTATTGAATTTTGGAGAACAGAAATAGAAATGTGAGTCCAATATATTCTATGGGCAGGCTCCACGAAATCAAATCAAATCATAAGAGAAAAAAGATACTTAAATGAAATACCGAGATTAGGGTAGTAGGATGCTGGTTCGAAAGTAATTGTATTACTTAATTATTACTCAATTAATATTAATTTAATTACAACCAAATCTTTAGAAATTCCATTTCTAGTTAGTAACTTCTATTGATTTTTTTGTTCTTTTCTTCTTCTTCGGTTCGGATCGAAAATAGAAGAATTTAAGTCGATCAAAAGGAGGTTCATGGCCAAGGGTAAGGATGTCAGAGTCAGAGTTATTTTGGAATGCGCCAGTTGTGTCCGAAATGGTATCAATAAAGAATTGCCGGGTATTTCTAGATATATTACTCAAAAGAGTCGACACAATACACCCAATCGATTAGAATTAAGAAAATTTTGTCGCTATTGTCGCACACATACGATTCATGGAGAAATAAAGA

**(TATTA)_6_ for BJ, (TATTA)_2_ for FD;**

**BJ:**

ATGATTTTTTCTCCTGTTCATCAAAGATTACTATTCCTTTTTTCCGGGTCTATTGGGACTCCTTTGTTTAGATTCATAAAAAGTATTGGACAAACAAAAAAACTTTTCCTGTTCTGACTGGTTGAAACCCAGATTAGATTTTTACGCATAAATCCTTTATGAATTTATTATCTTATCACAGTTTTATTTTCAATTTATTTTCTATTATATTCTATTTTTTCTATTATATTATATTATACAGTAAGTTTTATTTAGTTTAGTATAGAAAAAATTTTCAGGATCTCTCATCCGTAGAAATCCATAAAATAAATAAGAAATAAAATAATATAAAAATAATAATATATATTATATACACTAAATAATATATAAATAATTATTATTTTATTGGATTATCTTATCCACAAGATTCCTTCTTTCTTGGTAAGAATGAATATTCTGGAGAAATGACAGCGACTATGAATTAATCAATAGATTAAATTCTTCAAAAAAATTATAAGAAATAAAA

**FD:**

ATGATTTTTTCTCCTGTTCATCAAAGATTACTATTCCTTTTTTCCGGGTCTATTGGGACTCCTTTGTTTAGATTCATAAAAAGTATTGGACAAACAAAAAAACTTTTCCTGTTCTGACTGGTTGAAACCCAGATTAGATTTTTACGCATAAATCCTTTATGAATTTATTATCTTATCACAGTTTTATTTTCAATTTATTTTCTATTATATTCTATTTTTTCTATTATATTATACAGTAAGTTTTATTTAGTTTAGTATAGAAAAAATTTTCAGGATCTCTCATCCGTAGAAATCCATAAAATAAATAAGAAATAAAATAATATAAAAATAATAATATATATTATATATACTAAATAATATATAAATAATTATTATTTTATTGGATTATCTTATCCACAAGATTCCTTCTTTCTTGGTAAGAATGAATATTCTGGAGAAATGACAGCGACTATGAATTAATCAATAGATTAAATTCTTCAAAAAAATTATAAGAAATAAAA

**Polymorphic nuclear SSRs**

**nSSR_901**

**BJ:**

GAAGGGTGAAATTCCTTCTCTCCATTATTTTTGTTCCTAGATAAGATGCTTGCTACACTGATCATACATGTTACAGCCTTACAGGTGGATATATATGAACTGCACCAGCTGGTACATGAAATATTAGGAACAATGAGGAACATATATATGAGATCAAATAGGAACATAGGAGCTAGCAGGGCAGCAATATTGGTGATCTTGAGGGATGCATGGATGGATGGATGGATGGATGGATGGATGCAGCTAATTAATTAAGCCTCCAGTAGTAACATCTTGGCCTTGTTCATTTCCTTGAGCTCAATGAATTTCATGATATCCTGATCATCTCCGGCGGGCTTCTGATTCTCATGAGTCTGTAGATCCTTCAGCTCCTTCAACCAGATGTCGGCAAACTCACAGTTCTTGTAAGCCTCGAACCAGGGCCCCCCACAGGTATAATGTATGGCTTTTGGTAACGCCGTGTGGTCCTCCAACTTGTTGTGCCCAACCAGAAAGTTCCACGTGAAGGGGACTGACCCAATCTCCTCATCTTCCAACCACATGAACCTGCAAAACAATATGCATGGAAACGTACACGTACACCCACATTCAATTCATTCATTCATTCCTTCCTTCCTGTCCTTCATTAATTTGTCCGTACACGTACGTA

**FD:**

GAAGGGTGAAATTCCTTCTCTCCATTATTTTTGTTCATAGATAAGATGCTTGCTACACTGATCATACATGTTACAGCCTTACAGGTGGATATATATGAACTGCACCAGCTGGTACATGAAATATTAGGAACAATGAGGAACATATATATGAGATCAAATAGGAATATAGGAGCAGGGCAGCAATATTGGTGATCTTGAGGGATGCATGGATGGATGGATGGATGCAGCTAATTAATTAAGCCTCCAGTAGTAACATCTTGGCCTTGTTCATTTCCTTGAGCTCAATGAATTTGATGATATCCTGATCATCTCCGGCGGGCTTCTGATTCTCATGAGTCTGTAGATCCTTCAGCTCCTTCAACCAGATGTCGGCAAACTCACAGTTCTTGTAAGCCTCGAACCAGGGCCCCCCACAGGTATAATGTATGGCTTTTGGTAACGCCGTGTGGTCCTCCAACTTGTTGTGCCCAACCAGAAAGTTCCAGGTGAAGGGGACTGACCCAATCTCCTCATCTTCCAACCACATGAACCTGCAAAACAATATGCATGGAAACGTACACGTACACCCACATTCATTCATTCATTCCTTCCTTCCTTCCTTCCTTCCTGTCCTTGATTAATTTGTCCGTACCGTACGTA

**nSSR_1065**

**BJ:**

TCCTTGATCATACAAACACTTTGATTTGATAATAATTTATCATACTAAACCATAAATTCTTCTTAACTAATCATTGTCCTTGATTCAATCCAAAAGTTCATCTACATCAAATTCAAAACTAAATACTAAAACTATAATTTGGAATGGTAACCCTCTTTATAATGCCATGTTACGCACCATGTTATTATGCCACATCATCATCATCATGTTCACCCTCATAATCTCTCCATCTCATCACCATATCAATGTGCCATGTCATTGTTATGTTGTATTGTGCTCAATTGCAATAAACACAACTTTTAGGCATATTCAATAAGCTTTCAACCTTAATCTTAATTGGCTCTTCATAGGTCATCTTCATCTTCATCTTCTTGCAGCATCTCCTCCCAACTTGGCTCGTCTCTCATATCTTTCTTTAAAATATTTCGTTAATCATTGTCCTTTTAACATGATTTACATATGTAGTATTGATTTGAACTAGTTCACAACTAAATGTCTCATAATATAAGTTGAATTACTCTAAAAAGGACCCAAATAACAATTGATCCCTGTTTTGGCTGGTCTTCCTTATTCCATGTTGCTTATTGATAAGAAAAACTTTCTCTTCTTGAATTAGATGATCCTTTACACAACTGTGTTTAGGGTGCATAATTACATC

**FD:**

TCCTTGATCATATAAACACTTTGATTTGATAATAATTTATCATACTAAACCATAAATTCTTCTTAACTAATCATTGTCCTTGATTCAATCCAAAAGTTCATCTACATCAAATTCAAAACTAAATACTAAAACTATAATTTGGAATGGTAACCCTCTTTATAATGCCATGTTACGCACCATGTTATTATGCCACATCATCATCATGTTCACCCTCATAATCTCTCCATCTCATCACCATATCAATGTGCCATGTCATTGTTATGTTGTATTGTGCTCAATTGCAATAAACACAACTTTTAGGCATATTCAATAAGCTTTCAACCTTAATCTTAATTGGCTCTTCATAGGTCATCTTCATCTTCTTGCAGCATCTCCTCCCAACTTGGTTCGTCTCTCATGTCTTTCTTTAAAATATTTCGTTAATCATTGTCCTTTTAACATGATTTACATATGTAGTATTGATTTGAACTAGTTCACAACTAAATGTCTCATAATATAAGTTGAATTACTCTAAAAAGGACCCAAATAACAATTGATCCCTATTTTGGTTGGTCTTCCTTATTCCATGTTGCTTATTGATAAGAAAAACTTTCTCTTCTTGAATTAGGTGATCCTTTACACAACTGTGTTTAGGGTGCATAATTACATA

**nSSR_1163**

**BJ:**

GAATCCGTTAAACAGGCTTTCCTGTATTTCACTAACCCAAGCCATTTGTATCTTTACTATTGATTGCATCTAATCAGTTCCCATACATATCATTTACTTAACCTCTCCCTATGGCCTACAGACATGACTTCAATCCCATTATCTCATCACATGATTTCTCAACTTGTATGCAGGTTCTCACTATGCATACATAACAAACTATCAACAACCTCCATTGAAAGTAAGTAAGTAAAGGAGACTTATTGCTTGCATCTCCTTAGTTCCAAACCCTATACATGCATGCTTATTATTTGAACAACATCCATAAACAACATCAAGTCTTCCTAACCACATGCATCAACACTCAACCTTTACATCAACATAGGTTTATCCTTCCCACTCTCAAGCGCCTCTCTCTCTCTCAGCTTAATCGCCAACTGAACCTCAATGACAACACACAAACTCTAGTCCTATCATCAACCTTGCCTGGTGATTATCAACATACATGAAAACTCACAAACATGCAAGTATATGAGGCTTACCCAAACAACCCTTTGAAGCATGATAAAAATCACTAGGAAAATAGAAACCATGAATTTATAAACTCATTCCCAAAACAGACTGCAAG

**FD:**

GAATCCGTTAAACAGGTTTTCCTGTATTTCACTAACCCAAGCCATTTGTATCTTTACTATTGATTGCATCTAATCAGTTCCCATACATATCATTTACTTAACCTCTCCCTCTGGCCTACAGACATGACTTCAATCCCATTATCTCATCACATGATTTCACAACTTGTATGCAGGTTCTCACTATGCATACATAACAAACTATCAACAACCTCCATTGAAAGTAAGTAAGTAAAGGTAGACTTATTGCTTGCATCTCCTTAGTTCCAAACCATATACATGCATGCTTATTATTTGAACAACATCCATAAACAACATCAAGTCTTCCTAACCACATGCATCAACACTCAACCTTTACATCAACATAGGTTTATCCTTCCCACTCTCAAGCGCCTCTCTCTCTCTCTCAGCTTAATCGCCAATTGAACCTCAATGACAACACACAAACTCTAGTCCTATCATCAACCTTGCCGGGTGATTATCAACACACATGAAAACTCACAAACATGCATGTATATGAGGCTTACCCAAACAACCCTTTGAAGCATGATAAAAATCTCTAGGAAAATAGAAACCATGAATTTATAAACTCATTCCCAAAATAGACTGCAAG

**nSSR_1491**

**BJ:**

ACATAAACATGCGGCGTTGTCTTCTGAGCAACCACATGCAAAGAAGCTTCCTGATTTCCCAATATCAGCATCGGCAGTTAAGGGAGGAAGCAGAGGAGTACGGGGAGGGGGTTCAGTTCTCTTTTGACTTTAGCAGCACAGGTGAGTCGAAGACTCAGAGGTATGGAGGAGAGGAGTCCCCTTCTCGTACCCCAGGCTCCGGCATATGCGGAGAAGGGGCGCAGCAGCAGCAGCAGCAAGGCTACTGGCGCTGAGGAAGCCCCACCCAAGGACGCATTCCACATCGCGTACCTGATCTACTTCACGCTCGGCGCCGGATTCCTCCTGCCGTGGAACGCCTTCATCACCGCGATCGACTACTTCAGCTACCTCTACCCAGCGGCTCCCGTGGATCGCGTCTTCTCCGTATGCTATATGATATCCTGCTTGATCCCCCTTCTCTTCATAGTGGGACGGGGGGCACACCGCTCAAGCGTCTCCGCCAGA

**FD:**

ACATAAACATGCGGCGTTGTCTTCTGAGCAACCACATGCAAAGCTCCCTGATTTCCCAATATCAGCATCGGCAGTTAAGGGAGGAAGCAGAGGAGTACGGGGAGGGGGTTCAGTTCTCTTTTGACTTTAACAGCACAGGTGAGTCGAAGACTCAGAGGTATGGAGGAGAGGAGTCCCCTTCTCGTACCCCAGGCTCCGGCATATGCGGAGAAGGGGCGCAGCAGCAGCAGCAGCAGCAGCAGCAGCAAGGCTACTGGCGCTGAGGAAGCCCCACCCAAGGACGCATTCCACATCGCGTACCTGATCTATTTCACACTCGGCGCCGGATTCCTCCTGCCGTGGAACGCCTTCATCACCGCGATCGACTACTTCAGCTACCTCTACCCAGCGGCCCCCGTGGATCGCGTCTTCTCCGTATGCTATATGATATCCTGCTTGATCCCCCTTCTCTTCATCGTGGGATGGGGGGCACACCGCTCAAGCGTCTCCGCCAGA

**nSSR_2102**

**BJ:**

ACCTAGTACCGCGGCTGGGGTGATGGTGTAGGGAGAGTGCTATTGAGCAAATGCTTGATGGAGCGAGTCTTCGGCAGCTTTGGGGTCTGCATCTCACTTTTTTCAGCAGCTTTGGGACCTGCAGACTCCTGTTCAGAATGGTTGCATTTGATTTTTTCTTCCCGTCTGCGCTCCAGTGTTTCCTGCTTCTCTAAATGATAAGCACACAAGGAAAAAGATTTCTTCACATTAATGTTAACCTCTCCTGCTCACCTGATAGCACTGTGAGTTGTTGTGGGACCTTGCACTCCTGCACCCCTTCTTCACTCTACTATATAGATCATCAAACTGTCTTGTTTTCTCCAAAAGTCCTTCATGTTCATTGTCGAGATTGGCTGCAGACAAACATCTAGAAACAAAGTTAGGGTTCCTCCTCCTCCTCCCCCTGTTTGATCTTTTCCTCTTAGGTATGTTTCTTAATCTCTTCAGAGCCTCCTCTTCTTCAGAATCCAATATTAATATTGGTTCTTCATCGTAAACTTCAACAATTACATCATCATTTATAGGCACTTCATAGCATTGTTGGTTGAAAGCAGTTTGTTCTATTTGACCCCAGTTTATTGGCTTATTGAGGTCCAATATTTCACAATCTTCTTCTGCTTCAGAAAGCTCCATAGTTATTCCACC

**FD:**

ACCTAGTACCGCGGCTGCGGTGAAGGTGTAGGGAGAGTGCTCTTGAGCAAATGCTTGATGGAGCGAGTCTTCGGCAGCTTTGGGGTCTGCATCTCACTTTTTTCAGCAGCTTTGGGACCTGCAGACTCCTGTTCAGAATGGTTGCATTTGATTTTTTCTTCCCGTCTGCGCTCCAGTGTTTCCTGCTTCTCTAAATGATAAGCACACAAGGAAAAAGATTTCTTCACATTAATGTTAACCTCTCCTGCTCACCTGATTGCACTGTGAGTTGTTGTGGGACCTTGCACTCCTGCACCCCTTCTTCACTCTACTATATAGATCATCAAACTGTCTTGTTTTCTCCAAAAGTCCTTCATGTTCATTGTCGAGATTGGCTGCAGACAAACATCTAGAAACAAAGTTAGGGTTCCTCCTCCTCCTCCTCCTGTTTGATCTTTTCCTCTTAGGTATGTTTCTTAATCTCTTCAGAGCCTCCTCTTCTTCAGAATCCAATATTAATATTGGTTCTTCATCGTAAACTTCAACAATTACATCATCATTTATAGGCACTTCATAGCATTGTTGGTTGAAAGCAGTTTGTTCTATTTGACCCCAGTTTATTGGCTTATTGAGGTCCAATATTTCACAATCTTCTTCTGCTTCAGAAAGCTCCATAGTTATTCCACC
